# An autonomous power temperature sensor based on window-integrated transparent PV using sustainable luminescent carbon dots†

**DOI:** 10.1039/d3na00136a

**Published:** 2023-04-21

**Authors:** Sandra F. H. Correia, Lianshe Fu, Lília M. S. Dias, Rui F. P. Pereira, V. de Zea Bermudez, Paulo S. André, Rute A. S. Ferreira

**Affiliations:** a Instituto de Telecomunicações and University of Aveiro, Campus Universitário de Santiago 3810-193 Aveiro Portugal; b Department of Physics and CICECO – Aveiro Institute of Materials, University of Aveiro 3810-193 Aveiro Portugal rferreira@ua.pt; c Department of Electrical and Computer Engineering and Instituto de Telecomunicações, Instituto Superior Técnico, University of Lisbon 1049-001 Lisbon Portugal paulo.andre@lx.it.pt; d Chemistry Center and Chemistry Department, University of Minho 4710-057 Braga Portugal; e Chemistry Department and CQ-VR, University of Trás-os-Montes e Alto Douro 5000-801 Vila Real Portugal

## Abstract

The energy efficiency of buildings can be significantly improved through the use of renewable energy sources. Luminescent solar concentrators (LSCs) appear to be a solution for integrating photovoltaic (PV) devices into the structure of buildings (windows, for instance) to enable low-voltage devices to be powered. Here, we present transparent planar and cylindrical LSCs based on carbon dots in an aqueous solution and dispersed in organic–inorganic hybrid matrices, which present photoluminescent quantum yield values up to 82%, facilitating an effective solar photon conversion. These LSCs showed the potencial for being incorporated as building windows due to an average light transmittance of up to ∼91% and color rendering index of up to 97, with optical and power conversion efficiency values of 5.4 ± 0.1% and 0.18 ± 0.01%, respectively. In addition, the fabricated devices showed temperature sensing ability enabling the fabrication of an autonomous power mobile temperature sensor. Two independent thermometric parameters were established based on the emission and the electrical power generated by the LSC-PV system, which could both be accessed by a mobile phone, enabling mobile optical sensing through multiparametric thermal reading with relative sensitivity values up to 1.0% °C^−1^, making real-time mobile temperature sensing accessible to all users.

## Abstract 2

Sustainable luminescent solar concentrators based on carbon dots for window-integrated transparent photovoltaics as an autonomous power multiparametric mobile temperature sensor.

## Graphical abstract

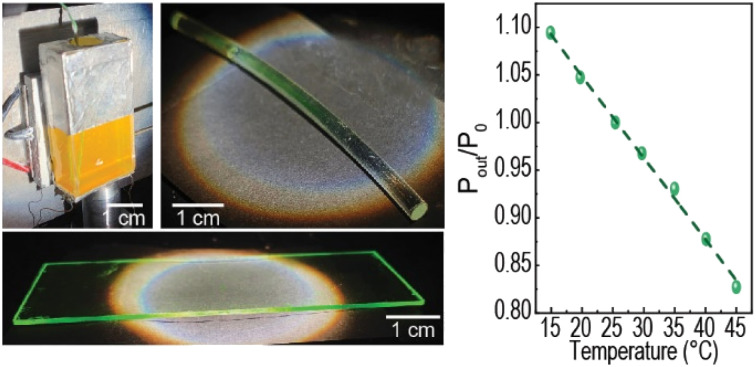

## 1 Introduction

Nowadays, the world's energy is mainly consumed by three sectors: building, industry, and transportation, in which the building sector represents the world's largest energy consumer.^1^ In the European Union (EU), energy management in buildings accounts for nearly 40% of the primary energy consumption (33% worldwide) and 36% of greenhouse gas (GHG) emissions.^2^ For the next decade, energy needs must also take into account the rise of Information and Communications Technologies (ICT), namely the deployment and full maturity of Internet of Things (IoT) systems, in which the industrial mass market associated is estimated at 350 billion dollars in 2025.^3^

In line with the United Nations, the EU promotes the construction of Zero Energy Buildings (ZEB)^4^ as the new building target. This solution has received increasing attention in recent years, and has become part of the energy policy in several countries. Buildings' energy performance has been a top priority concern in the EU. Three European directives stimulate the transition to decarbonized buildings: (1) the 2020 Climate and Energy Package,^5^ associated with the Energy Efficiency Directive,^6^ which targets a 20% cut in GHG emissions (from 1990 levels), 20% energy from renewables, and 20% improvement in energy efficiency; (2) the 2030 Climate and Energy Framework,^7^ which targets 40% cuts in GHG emissions (from 1990 levels), a 32% share for renewable energy, and 32.5% improvement in energy efficiency; and (3) the 2050 Long-Term Strategy,^8^ which targets climate-neutrality by 2050 and net-zero GHG emissions. All these targets are aligned with the Paris Agreement on climate change and the European Green Deal.^9^

Solar energy is the dominant renewable source that is, however, unable to cope with all the energy needs.^10^ A population increase in large metropolises leads to the construction of taller buildings, where the contribution of the roof-mounted photovoltaic (PV) devices to the energy needs decreases with the number of floors. Additional energy may be obtained through urban PV panels on façades through luminescent solar concentrators (LSCs) that appear as a strategy to integrate solar-harvesting devices into buildings.^11^ LSCs consist of planar waveguides doped or coated with emissive materials. Sunlight is absorbed by the materials and re-emitted at distinct wavelengths matching the PV cell operating spectral region. The emitted light is guided by total internal reflection towards PV cells coupled to the edges, where it is converted into electricity. LSCs operate under direct or diffuse conditions, and are highly transparent, providing high design freedom that may be applied over large areas and incorporated into facades or windows, as they operate similarly under direct and diffuse sunlight.^12^ Materials with absorption in the ultraviolet (UV)/blue spectral region have been sought, as they result in transparent and colourless waveguides, ideal for windows, contributing to aesthetics and avoiding distortion of the spectrum of the natural light, which typically happens in highly efficient luminophores with significant absorption in the visible region.^13,14^ Examples include organic synthetic dyes, quantum dots (QDs), lanthanide ions, and organic molecules from natural renewable sources.^15–17^ In particular, QDs present excellent optical properties (*e.g.*, size, shape, chemical composition, tuneable absorption spectra, structurally engineered Stokes shift, high photoluminescent quantum yield (*ϕ*) values, and enhanced photostability) compared to conventional organic dyes and have attracted a lot of attention as fluorophores for LSCs.^18–21^ However, most inorganic QDs (*e.g.*, PbS/CdS, CdSe/CdS, and CuInSe/ZnS) are very sensitive to oxygen and moisture during the synthesis, purification, and LSC preparation process,^21^ and some contain toxic elements (*e.g.*, Cd and Pb).^22^ Very recently, less toxic or even non-toxic luminescent nanomaterials, such as carbon dots (CDs), have emerged as alternatives to colloidal QDs. CDs exhibit unique properties, such as tuneable optical features, moderate *ϕ* values (30–78%) compared to conventional organic dyes (>90%), photostability, and a large molar absorption coefficient, which are suitable for capturing solar radiation, thus highlighting their great potential as fluorophores for LSCs, as they may significantly enhance the device performance.^17,23–25^ Moreover, because of their excellent optical performance combined with relative innocuity, easy preparation, and cost-effectiveness, CDs have exceptional advantages for the development of a new type of metal-free material.

A recently reported approach showed the importance of including additional sensing abilities for LSCs to behave as sunlight-powered optical temperature sensors.^16^ Luminescence is a remote method for temperature measurement based on the thermal dependence of the phosphor emission combining high relative thermal sensitivity (*S*_r_ > 1.0% °C^−1^) and spatial resolution (10^−6^ m) with short acquisition times (<10^−3^ s).^26–28^ Very recently, this concept originally formulated to be used with conventional spectrometers was extended to mobile optical sensing (mOptical) in which the sensing function is mediated by the charge-coupled-devices (CCDs) of smartphones.^29,30^

In this work, we report the fabrication of a novel window with high color rendering index (CRI = 97) made of an integrated LSC-PV system incorporating an organic–inorganic hybrid matrix doped with Rhodamine B (RhB)-derived CDs with the ability to be autonomously powered by the Sun and working as a mobile temperature sensor with *S*_r_ = 1.0% °C^−1^ based on multiparametric thermal reading (multi-readout concept). This particularity will allow the fabrication of a sensor with a self-test function to assess any possible malfunctions or need for calibration. The thermometric parameters allow access to temperature through the use of a mobile phone fostering the popularization of luminescence thermometry in real-world applications and featuring mOptical sensing.

## 2 Experimental section

### 2.1. Materials and methods

RhB (Sigma, >95%), α,ω-diaminepoly(oxyethylene-co-oxypropylene) (ED-600, Huntsman), 3-isocyanatopropyltriethoxysilane (ICPTES, 95%, ABCR), sodium hydroxide (NaOH), hydrochloric acid (HCl) and ethanol (EtOH) were used without purification.

#### 2.1.1 Synthesis of the CDs

The CDs derived from RhB were synthesized according to a method previously reported.^31–33^ Briefly, 96 mg of RhB was dissolved in 15 mL of a 0.67 M NaOH solution with ultrasonic treatment. The resulting clear solution was transferred to a poly(tetrafluoroethylene)-lined autoclave (20 mL) and heated at 180 °C for 8 h. To obtain the solid CDs, a volume of 5 mL of 0.01 M HCl was added to the above solution under stirring, followed by the dropwise addition of 2 mL of acetic acid. The precipitate formed was filtered, washed with water, and dried at 60 °C. CDs aqueous solution (0.05 mg mL^−1^) was obtained by the addition of 0.8 mg of solid CDs to 16 mL of distilled water.

#### 2.1.2 Synthesis of the d-U(600)/CDs composites

The urea cross-linked organic–inorganic hybrid precursor, d-UPTES(600), was first synthesized according to the procedure reported in the literature.^34^ Then, different volumes of the CDs solution (25, 50, 100, or 200 μL) were added to 1.0 g of d-UPTES(600) separately, followed by the addition of 1.0 mL of EtOH. The mixtures were stirred at room temperature to yield homogeneous solutions. The resulting sols were maintained at 70 °C for several days to produce the dU6/CDs composites. These were denoted as dU6/CDs-1, dU6/CDs-2, dU6/CDs-3, and dU6/CDs-4 for 25, 50, 100 and 200 μL doping, respectively (Table S1 in the ESI†).

#### 2.1.3 Fabrication of LSC based CDs aqueous solution

The planar LSC was fabricated using an optical glass cuvette (CM Scientific) with dimensions tailored to fit the PV cell surface to which the above-mentioned CDs aqueous solution was transferred. The optical power at the LSC output was determined by illuminating the top surface of the LSCs (2.0 × 2.0 cm^2^) with AM1.5G illumination from a solar simulator (Model 10500, Abet Technologies). The optical power at the LSC output was estimated using a commercial c-Si PV panel (KXOB22-01X8F, IXYS) coupled to one edge of the LSC (2.0 × 1.0 cm^2^), while the remaining edges were covered with reflective tape ensuring the back-reflection of the light. An alternative configuration could consider the use of PV cells in all the LSC edges (see the ESI† for details). The PV panel was attached taking advantage of the adhesive side of the reflective tape.

#### 2.1.4 Fabrication of LSCs based on the dU6/CDs

Typically, a mass of 7.0 g (6.40 mmol) of d-UPTES(600) was mixed with 7.0 mL of EtOH (d-UPTES(600) : EtOH = 1 : 1 w/v) under stirring to yield a homogeneous solution. Then 0.69 mL (38.40 mmol) of HCl acidified water (pH = 2) was added (molar ratio d-UPTES(600) : H_2_O = 1 : 6) under stirring to catalyse the hydrolysis and polycondensation reactions. Finally, 0.70 mL of the CDs NaOH solution was added, and the resulting sol was stirred at room temperature for 2 h. The glass substrates were ultrasonically washed in Hellmanex III washing solution for 30 min, rinsed with water, and dried in an air atmosphere. The luminescent films were deposited on the substrate by the spin-coating technique by casting 0.8 mL of the above sol onto the substrate (25 × 75 mm^2^) and using a consecutive process of 600 rpm (10 s), 800 rpm (10 s), and 1000 rpm (20 s) (SPIN150 spin-coater). The obtained luminescent films on the substrate were placed in the air. The optical power at the LSC output was estimated using the above-mentioned c-Si PV panel with a mask matching the LSC edge area dimensions (25.0 × 1.0 mm^2^). The fibre-based LSCs were fabricated by using 5.0 × 10^−2^ m segments of PMMA-based POFs (Jiangsu TX Plastic Optical Fibers Co., Ltd.) with a refractive index of ∼1.49 in the visible spectral region and a diameter of 3.0 × 10^−3^ m. Then, the fibre segments were vertically immersed in the sol at a velocity of 1.6 × 10^−3^ m s^−1^ using a homemade dip-coating system. The electrical output of the fiber-based LSC was estimated using a commercial photodiode (IF D91, Industrial Fiber Optics, Inc.) to accommodate the shape of the fiber, which may lead to lower conversion efficiency. In both cases, the coupling between the LSCs and the PV panel/photodiode was attached to each other using mechanical fixtures, optimizing the positioning of the LSC to maximize the amount of light that is captured and concentrated onto the PV panel/photodiode.

### 2.2. Structural characterization

Powder X-ray diffraction (XRD) patterns were recorded in the 2*θ* range of 3.0 to 60.0° on a Panalytical Empyrean diffractometer under CuKα irradiation (*λ* = 1.5418 Å) at room temperature. The Fourier transform infrared (FT-IR) spectra for all the materials were acquired on a Bruker Optics Tensor 27 spectrometer (Bruker Corporation, Billerica, MA, USA) using the KBr pellet technique with 64 scans and 2 cm^−1^ resolution. Transmission electron microscopy (TEM) images were obtained at the Iberian Nanotechnology Laboratory using a JEOL JEM 2100 (200 kV) microscope. Samples were dispersed in water and placed into the analysing grids (UC-A on holey 400 mesh Cu grids, Ted Pella ref. 01824) by drop-casting, followed by drying at room temperature. X-ray photoelectron spectroscopy (XPS) was performed using a ESCALAB 250Xi (Thermo Fisher Scientific) with monochromated AlKα (1486.68 eV) radiation, operated at 220 W and 14.6 kV, with a spot size of 650 μm. XPS spectra were collected at pass energies of 100 eV and 40 eV, for survey spectra and individual elements, respectively. The energy step for individual elements was 0.1 eV. Samples were mounted on the sample holder using double-sided adhesive carbon tape. XPS spectra were peak-fitted using Avantage data processing software (Thermo Fisher Scientific) and the Shirley-type background was used. All the XPS peaks are referenced to the adventitious carbon C1s C–C peak at 284.8 eV. Quantification was done using sensitivity factors provided by the Avantage library. Charge neutralization was achieved with both low energy electron and argon ion flood guns (<0.1 eV, 120 μA and 70 μA current respectively).

### 2.3. Optical characterization

The photoluminescence spectra were recorded with a modular double-grating excitation spectrofluorimeter with a TRIAX 320 emission monochromator (Fluorolog-3, Horiba Scientific) coupled to an R928 Hamamatsu photomultiplier. The emission spectra were recorded in the temperature range of 25–50 °C with a step of 5 °C. To have a calibration curve independent of the power fluctuations of the solar simulator, the thermometric parameter values were normalized to the ones at 50 °C. The temperature was increased with a homemade Peltier system (0.1 °C accuracy) and recorded using an immersed thermocouple (0.1 °C accuracy, K-type, VWR). To ensure that the solutions reached the steady-state temperature, a time interval of 300 s was allowed between consecutive temperature measurements. The *ϕ* values were measured at room temperature using a system (C9920-02, Hamamatsu) with a 150 W xenon lamp coupled to a monochromator for wavelength discrimination, an integrating sphere as the sample chamber, and a multichannel analyzer for signal detection. The method is accurate to within 10%. Optical parameters of the transmitted light were recorded using an integrating sphere (ISP 150 L, Instrument Systems) connected to a detector (MAS40-121, Instrument Systems), with an integration time of 10 s. Temperature-dependent emission spectra of the CDs aqueous solution were obtained when the solution was irradiated with an AM1.5G solar simulator (Model 10500, Abet Technologies). The detection system to collect the emission spectra uses an optical fibre connected to a portable spectrometer (FX Ocean Optics) for real-time acquisition. The temperature dependence of the electrical parameters delivered by the LSC-PV system was measured in the temperature range of 15–45 °C with a step of 5 °C. In this case, the thermometric parameter values were normalized to those at 45 °C. The temperature was increased with the above-mentioned Peltier system. The temperature-dependent emission spectra of the samples were used to assess their thermometric performance as luminescent thermometers. The data analysis was carried out by using a custom script written in MATLAB 2022a under the license provided to the University of Aveiro. In the initial step, a baseline correction was applied to each emission spectrum to remove the electrical noise arising from the spectrometer signal. The integrated emission intensities were then obtained by integrating the baseline-corrected emission spectra at the indicated spectral regions. The spectral noise was calculated as the difference between the original emission spectra and the smoothed spectra (smooth function in MATLAB, span = 4), and the histograms of the noise values were adjusted to a Gaussian function (*r*^2^ > 0.98) centered at zero. The noise of each emission spectrum was then defined as the standard deviation of its corresponding Gaussian fit. The signal-to-noise ratio (SNR) was defined as the maximum intensity of each spectrum divided by the calculated noise. Finally, the uncertainties of the integrated areas were obtained by dividing them by their SNR. The figures of merit to evaluate the performance of the thermometers are the *S*_r_ and the temperature uncertainty, *δT*, defined as:^28,35^1
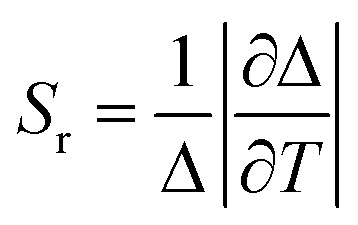
and2
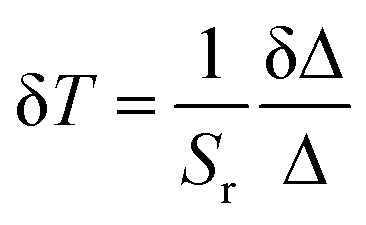
where δ*Δ* is the uncertainty in the determination of the thermometric parameter *Δ*.

## 3 Results and discussion

### 3.1. CDs structural and optical characterization

**Fig. 1:**
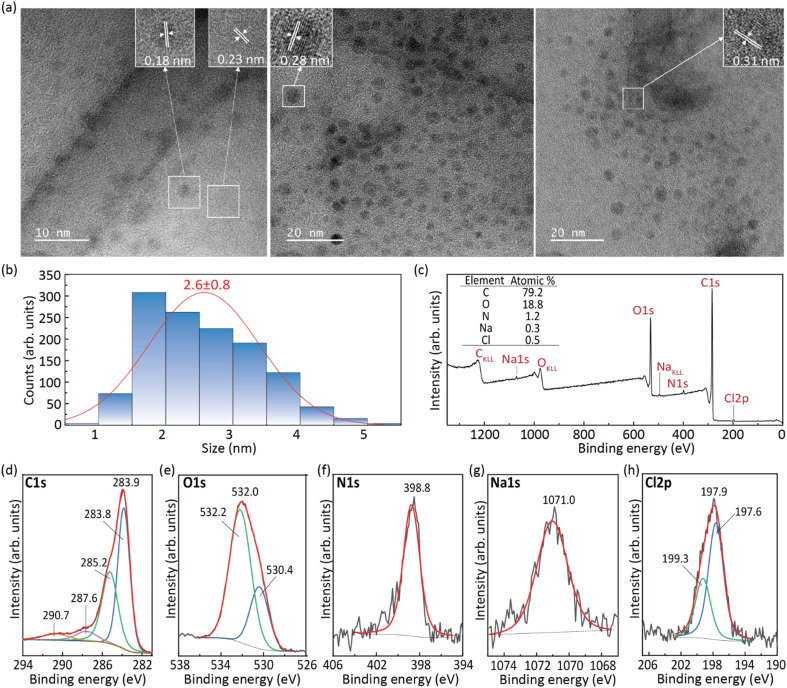
(a) TEM images, (b) size distribution, (c) XPS survey spectrum and high resolution XPS spectra of (d) C 1s, (e) O 1s, (f) N 1s, (g) Na 1s and (h) Cl 2p of the CDs. Insets in (a) reproduce the high resolution TEM images of individual CDs showing lattice fringes with the corresponding d-spacing determined by FFT analysis. Insets reproduce the (a) high resolution TEM images of individual CDs showing lattice fringes with the corresponding *d*-spacing determined by FFT analysis, and (c) atomic % of elements.

The CDs obtained from the hydrothermal reaction of RhB in the presence of NaOH were examined by TEM (Fig. 1a and b) and XPS (Fig. 1c–h). The results of the XRD and FT-IR analyses of the CDs, pure RhB, and the hybrids dU6/CDs-*x* (*x* = 1, 2 3, and 4) can be found in Fig. S1a and S1b,† respectively.

The representative TEM image of the CDs shown in Fig. 1a demonstrates that the CDs synthesized exhibit a uniform and nearly spherical shape. Their average diameter is 2.6 ± 0.8 nm, with sizes ranging from 0.8 to 6.0 nm (Fig. 1b). High-resolution TEM measurements (insets of Fig. 1a) revealed well-resolved lattice fringes with interplanar spacings of 0.18, 0.23, 0.28 and 0.31 nm which correspond to the (103), (021), (111), and (110) diffraction planes of orthorhombic graphitic carbon belonging to the *Pban* space group (*a* = 4.0480 Å, *b* = 4.8850 Å, *c* = 6.4950 Å and *α* = *β* = *γ* = 90°),^36^ respectively.

XPS analysis was performed to gain insight into the surface composition of the CDs. The full-range XPS survey spectrum shows three characteristic binding energy peaks at 283.9, 398.8, and 532.0 eV (Fig. 1c) assigned to C 1s, O 1s, and N 1s. Additionally, peaks attributed to Na 1s (1071.0 eV) and Cl 2p3 (197.9 eV) can also be identified, indicating the presence of sodium, presumably as a residue of the synthetic procedure, which involved the use of NaOH, and chlorine, the source of which could be RhB or the HCl used in the synthesis. Auger peaks of C_KLL_, O_KLL_, and Na_KLL_ are also observed in the same spectrum. As expected, quantitative analysis of this XPS spectrum led to an atomic predominance of carbon (79.2%) followed by oxygen (18.8%) and nitrogen (1.2%), and residual percentages, sodium (0.3%) and chlorine (0.5%) (inset of Fig. 1e). The high-resolution XPS spectrum of C1s was deconvoluted into four components at 283.8, 285.2, 287.6 and 290.7 eV (Fig. 1d), tentatively assigned to C=C, C–H/C–N, C=O/C–O, and π–π* bonds, respectively.^37–40^ The shape of the peaks at 283.8 and 290.7 eV is indicative of the presence of hybridized carbon atoms to form sp2 hybrid orbitals of the trigonal structure of graphite. This finding corroborates the conclusions from the analysis of HR-TEM images since the determined interplanar spacings found were consistent with the occurrence of graphitic carbon. The presence of O-containing bonds in the carbon structure demonstrates the existence of anchoring sites in the graphene periphery (*e.g.*, ketone and ether groups) for interaction with other molecules and thus functionalization. The high-resolution XPS spectrum of O 1s was deconvoluted into two peaks at 532.2 and 530.4 eV (Fig. 1e), assigned to C–O bonds and physically adsorbed oxygen, respectively.^37,39–41^ The high-resolution XPS spectrum of N 1s is dominated by a peak at 398.8 eV (Fig. 1f), suggesting not only that N is predominantly available in the pyridinic form, but also that the N atoms are mainly located in the graphene sheet edge.^39,42^

**Fig. 2:**
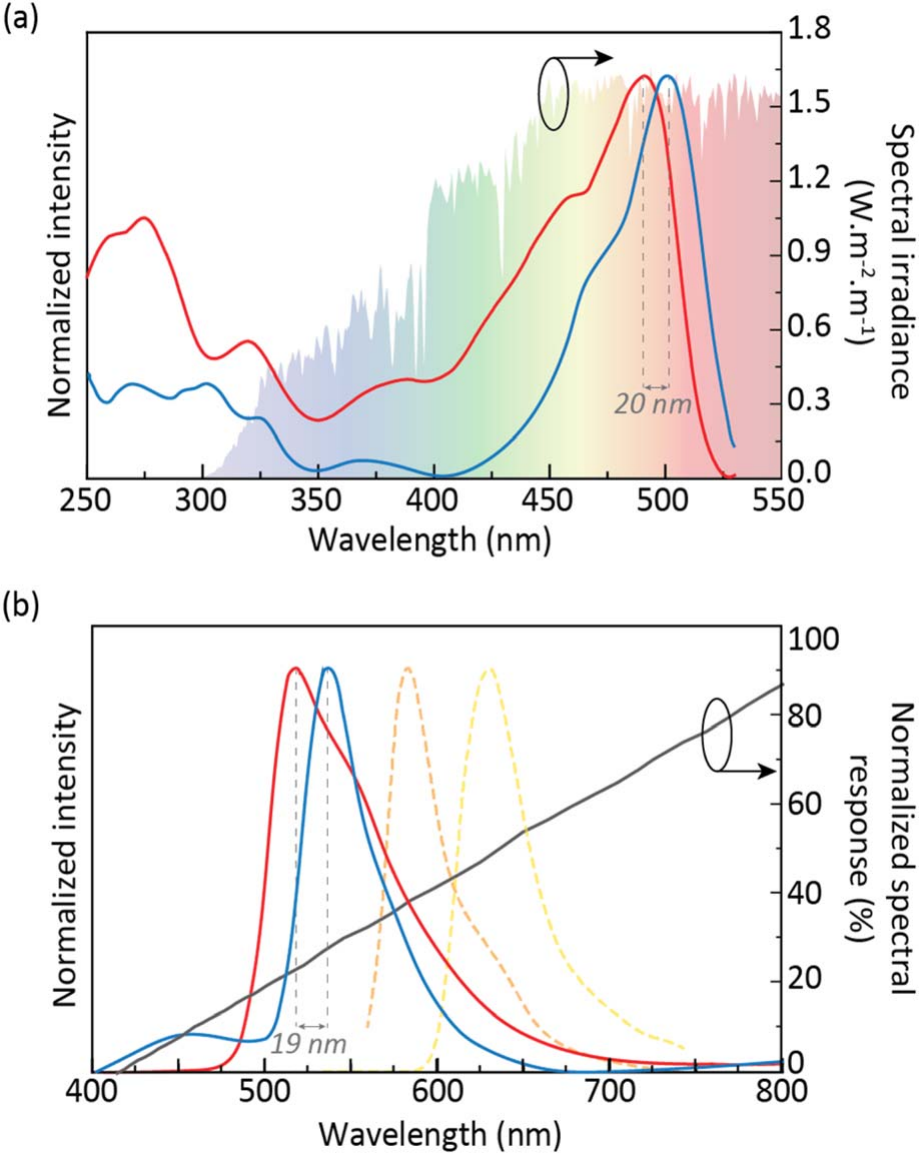
(a) Excitation and (b) emission spectra of the CDs aqueous solution (red line) and the dU6/CDs-1 (blue line) monitored at 550 nm and excited at 360/370 nm, respectively. The emission spectra of RhB aqueous solution at concentrations of 10^−3^ (yellow dashed lined) and 10^−5^ M (orange dashed line) are also presented. The shadowed area in (a) is the AM1.5G solar irradiation spectrum. The c-Si spectral absorption is shown on the right *y*-axis of (b).

The CDs aqueous solution photoluminescence features revealed an excitation spectrum that broadly overlaps the solar spectrum (Fig. 2a, red line) due to a component centered at 491 nm. For a given concentration, the emission spectrum shows an excitation wavelength-independent feature (Fig. 2b, red line, and S4 in the ESI†). This band arises from the molecular state,^43,44^ which was formed by fluorophore moieties on the graphitic carbon core.^33^ The maximum *ϕ* value found for the CDs aqueous solution was 82 ± 8% (488 nm excitation), ensuring easy excitation under solar irradiation and low-power light sources, such as LEDs, enabling emission quantification through smartphone CCD cameras.^30^ It should be noted that the *ϕ* values of the CDs aqueous solutions are much higher than those of RhB aqueous solutions (Table S2† in the ESI). As the concentration of the CDs increases, the emission wavelength shifts from around 515 to 535 nm (Fig. S3 in the ESI†). It is also noted that upon comparing the emission spectra of the CDs to that of the RhB aqueous solution, a blue-shift is observed. Although the emission peak of RhB is concentration-dependent due to the formation of *J*-type agglomerates,^45^ a significant deviation from the CDs emission peaks (114 and 66 nm for the 10^−3^ and 10^−5^ M RhB aqueous solutions, respectively) can be observed (Fig. 2b and S2 in the ESI†),^33^ indicating the absence of RhB molecules in the final CDs.

To enlarge the application range of CDs, incorporation into solid matrices is an important step. In this sense, CDs were incorporated into the di-ureasil organic–inorganic hybrid matrix d-U(600). The photoluminescence features of the dU6/CDs samples revealed a red-shift of both emission and excitation spectra relative to those of the CDs aqueous solution of around ∼20 nm, peaking at 510 and 535 nm, respectively (Fig. 2a, blue line, and Fig. S5–S8 in the ESI†) which is due to the polarization interaction between CDs and the di-ureasil matrix, with similar results (red-shift on the emission peak) found in the literature when 4-hydroxy-2-methyl-1,5-naphthyridine-3-carbonitrile^46^ or Safranine-O was incorporated into d-U(600).^47^ It is worth noting that the least concentrated dU6/CDs sample revealed a prominent band in the blue spectral region, arising from the hybrid's emission.^34^ The *ϕ* values were evaluated in the 270–510 nm range, yielding maximum *ϕ* value of 78 ± 8% under 510 nm excitation, similar to that of the CDs aqueous solution (Table S1† in the ESI), thus ensuring efficient sun power conversion. As this is the sample that presented the highest *ϕ* value for the lowest CDs concentration (0.43 mg mL^−1^), it was selected in subsequent studies. Therefore, hereafter in this manuscript, this sample will be simply denoted as dU6/CDs.

### 3.2. Performance of the LSCs based on CDs

**Fig. 3:**
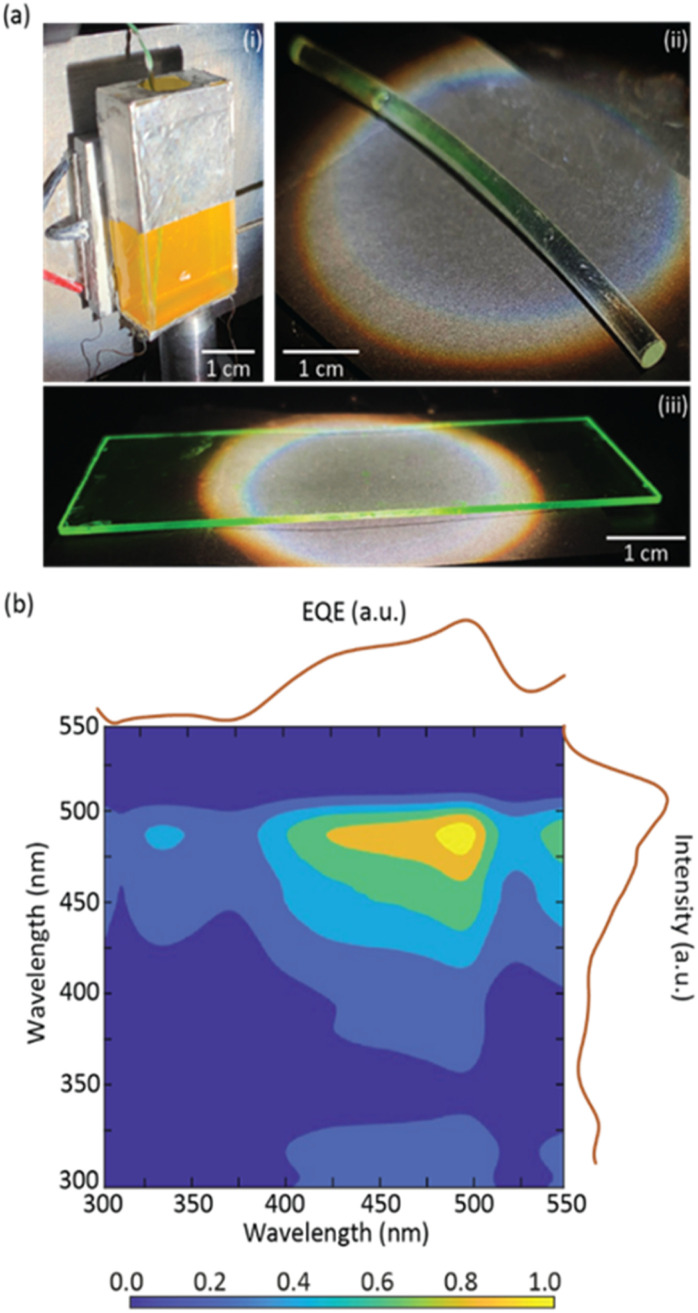
(a) Photographs of (i) the fabricated CDs aqueous solution planar LSC and (ii) the cylindrical and (iii) planar LSCs based on dU6/CDs under AM1.5G irradiation. (b) Cross-correlation between the EQE of the LSC-PV system based on the CDs aqueous solution and its excitation spectrum.

The CDs aqueous solution was used to fabricate a planar LSC by filling a glass container coupled to a c-Si PV cell. The device was characterized under simulated AM1.5G irradiation (Fig. 3a) and the optical conversion efficiency (*η*_opt_ = *P*_out_/*P*_in_, where *P*_out_ is the output optical power and *P*_in_ is the incident optical power) and power conversion efficiency (PCE = *P*^el^_out_/*P*_in_, where *P*^el^_out_ is the electrical output power) were calculated (see the ESI† for details). This device yielded *η*_opt_ and PCE values of 5.4 ± 0.1% and 0.18 ± 0.01%, respectively, which are comparable to the values found in the literature for CD-based LSCs (Table 1).

**Table 1:** Overview of reported LSCs incorporating CDs as optically active centers^a^

Fluorophore/host	Dimension (cm^3^)	*η*_opt_ (%)	PCE (%)	Ref.
RhB-CDs/Water	2.0 × 2.0 × 1.0	5.43	0.18	This work
RhB-CDs/d-U(600)	3.5 × 2.5 × 0.1	0.058	8.3 × 10^−4^	
RhB-CDs/d-U(600)	*L* = 3.5; *D* = 0.3	1.7	0.014	
N-CDs/PMMA	2.5 × 1.6 × 0.1	4.75	3.94	52
N-CDs/PMMA	2.0 × 2.0 × 0.2	12.23	2.63	53
Si-CDs/Ormosil	3.0 × 3.0 × 0.3	12		54
N-CDs/PVP	1.8 × 1.8 × 0.1	5.02	4.97	55
CDs/PVP-CdSe/CdS/PLMA	10 × 10 × 0.2	1.4	—	21
CDs/PVP; Perovskite QDs/PLMA-co-EGDA	10 × 10 × 0.2	—	3.05	56
CDs-OLA/PLMA	10 × 1.5 × 0.2	1.2		
N-CDs/PDLC	5.0 × 2.5 × 0.4	4.52	2.49	57
CDs/PVP	10 × 10 × 1.0	0.92		58
CDs/PVP-TPFE-Rho/PMMA	2.5 × 2.0 × 0.3		4.06	59
CDs/PVP	10 × 10 × 0.9	1.6	0.7	60
Si-CDs/siloxane	3.0 × 3.0 × 0.3	3.9		61
b-CD/PVA; g-CD/PVP; r-CD/PVP + PEI b	8 × 8 × 0.8	2.3		62
y-CDs/PVP	2.5 × 2.5 × 0.1	3.51	2.39	63
g-CDs/PVP		2.76	1.94	
r-CDs/PVP		2.77	1.96	
g-CDs/y-CDs/r-CDs/PVP b	2.5 × 2.5 × 0.3	4.03	2.92	
Si-CDs-D1	2.5 × 2.5 × 0.1	7.58	6	64
Narrow sized CDs/PVP	15 × 15 × 0.5	2.2	1.13	65
r-CDs/PVP	10 × 10 × 0.7		1.9	66
g-CD/PVP			1.7	
r-CD/g-CD/PVP b	10 × 10 × 1.4		2.3	
y-CDs/PVP	10 × 10 × 0.9	2.6	2.3	67
r-CDs/PVP		3	2.7	
y-CDs/r-CDs/PVP ^a)^	10 × 10 × 0.2	4.3	3.8	
CDs/Ag/SiO_2_	3.0 × 2.0 × 0.2	1.23	0.43	68
CDs/Ag/SiO_2_/PVP	5.0 × 5.0 × 0.2	0.9	0.62	
b-CDs/g-CDs/PNIPAm	2.5 × 2.5 × 0.2	2.7		69
y-CDs/p(MMA-NIPAm)		5.84		70
Ba^2+^-capped CDs/PVP	10 × 10 × 0.4	3.2	1.9	71
Ca^2+^-capped CDs/PVP		2.9	1.7	
Si-CDs/PVP	15 × 15 × 0.5		2.06	72
Si-CDs/PVP	5.0 × 5.0 × 0.5	4.8	4.36	
OSi-CDs	2.5 × 2.5 × 0.1	4.5	0.12	73
OSi-CDs		5.89	0.16	
OSi-CDs	5.0 × 5.0 × 0.1	3.13	0.06	
DAMO-CDs/PMMA	2.0 × 2.0 × 0.5	9.3		74
y-CDs/PVP	10 × 10 × 1.0	4.56	4.1	75
BWCDs/HEMA/EGDMA	2.5 × 2.5 × 0.15	1.36		76
g-QDs/EVA	2.5 × 2.5 × 0.2	3.08		
r-QDs/EVA		2.55		
BWCDs/HEMA/EGDMA;g-QDs/EVA b		1.89		
BWCDs/HEMA/EGDMA;r-QDs/EVA b		2.54		
BWCDs/HEMA; g-QDs/EVA; r-QDs/EVA b		3.76		

a *L* – length; *D* – diameter; N-CDs – nitrogen-doped carbon dots; Si-CDs – silicon-doped carbon dots; y-CDs – yellow-emitting carbon dots; b-CDs – blue-emitting carbon dots; g-CDs – green-emitting carbon dots; r-CDs – red-emitting carbon dots; OSi-CDs – organosilane carbon dots; BWCDs – blue-white emitting carbon dots; PDMS – polydimethylsiloxane; PMMA – poly(methylmethacrylate); PVP – poly(vinylpyrrolidone); PEI – poly(ethyleneimine); OLA – oleylamine; PLMA – poly(lauryl methacrylate); EGDA – Ethylene glycol diacetate; PDLC – Polymer-dispersed liquid crystals; PNIPAm – Poly(N-isopropylacrylamide); DAMO – N-[3-(trimethoxysilyl)propyl]ethylenedi-amine; HEMA – hydroxyethyl methacrylate; EGDMA – ethylene glycol dimethacrylate.

b Tandem LSC.

To demonstrate the processability and applicability of the dU6/CDs material, planar and cylindrical LSCs (Fig. 3a) were fabricated by coating a glass substrate and a plastic optical fiber (POF). In both cases, it is possible to observe a bright green emission that is guided and concentrated at the edges of the substrates ensuring transparency of the surface. These LSC-PV systems yielded *η*_opt_ and PCE values of 0.058 ± 0.003% and (8.34 ± 0.01)×10^−4^%, respectively, for the planar LSC, and 1.70 ± 0.09% and (1.40 ± 0.01) × 10^−2^%, respectively, for the cylindrical LSC (Table 1), proving the suitability of this approach to fabricate LSCs with distinct geometries. Moreover, the planar LSC-based dU6/CDs was kept under ambient conditions, and, as we write, the LSC still retains ∼90% of its *η*_opt_ after 9 months, indicating that the LSCs have good photostability.

External quantum efficiency (EQE) measurements are an indicator of the number of incident photons of a specific wavelength arriving at the PV cell. An increase in EQE when illuminating the LSC with the CDs aqueous solution excitation wavelengths indicates that the light arriving at the PV cell has a strong contribution from the CDs emission. It is shown that EQE measurements showed a good correlation with the excitation spectra of the CDs aqueous solution, demonstrating that the photons arriving at the PV cell are indeed originating from the CDs emission, with maximum value of ∼3.7% (Fig. 3b and S10† in the ESI).

One of the main applications of LSCs is their use as building windows, and thus LSC devices should not distort the spectrum of the natural light to ensure that the transmitted light meets basic indoor activity requirements.^48^ The transmitted light is often assessed by the Average Visible Transmission (AVT) parameter, which is dependent on the photopic response of the human eye:^49–51^3
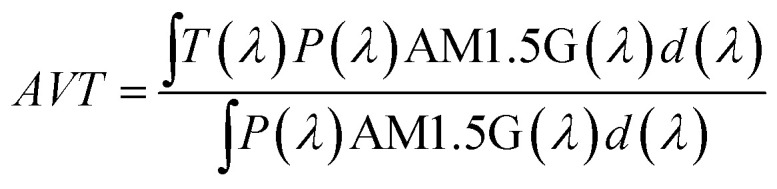
where *T*(*λ*) is the transmission spectra (Fig. S11† in the ESI), P(*λ*) is the photopic response of the human eye and AM1.5G(*λ*) is the solar photon flux spectrum (photons per s m^−2^). This parameter gives a measure of how much light people will perceive as passing through the LSC. The AVT of the fabricated planar LSCs was found to be 91% (dU6/CDs) and 63% (CDs aqueous solution), which lie in the range between 55% and 90%, considered acceptable for window applications.^49,50^

To quantify the color appearance of the planar LSCs and its effects on color perception, the transmitted light was analysed in terms of the Commission Internationale de l’Éclairage (CIE) 1931 color space diagram coordinates and color rendering index (CRI) which evaluated the ability to accurately render the color of objects. The light transmitted through the LSCs based on dU6/CDs showed CIE (*x*,*y*) coordinates (0.34, 0.34) while the LSC based on the CDs aqueous solution showed coordinates of (0.39, 0.41) which are very close to ideal white light coordinates (0.33, 0.33). The CRI is evaluated on a 0 to 100 scale, with a CRI above 70 being considered good quality and CRI above 95 of excellent quality.^49,77^ In the case of the fabricated LSCs, CRI values of 97 and 91 were found for the planar LSCs based on dU6/CDs and CDs in aqueous solution, respectively, revealing non-distortion of incoming sunlight (Fig. S12† in the ESI). Reinforcing it, the color correlation temperature (CCT) values of the transmitted light (5145 K for the dU6/CDs LSC) is very close to that of the incoming light (5161 K). In the case of the LSC based on CDs aqueous solution, the transmitted light revealed a CCT of 3855 K, which is adequate for residential or commercial places, revealing a high level of suitability as solar windows.

### 3.3. CDs LSC as a temperature sensor for IoT

**Fig. 4:**
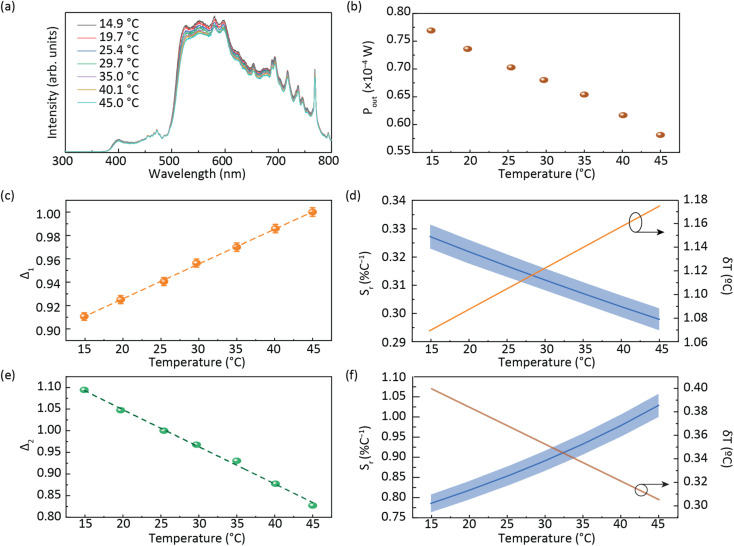
(a) Emission spectra of the planar LSC based on CDs aqueous solution under AM1.5G and (b) electrical power generated by the LSC-PV system as a function of the temperature. A temperature calibration curve with the thermometric parameters (c) *Δ*_1_ and (e) *Δ*_2_ (the lines are the best linear fit with *r*^2^ > 0.99) and (d) relative thermal sensitivity *S*_r_ calculated using eqn (1) and temperature uncertainty δ*T* calculated using eqn (2) for *Δ*_1_ and (f) *Δ*_2_.

Fig. 4a shows that the emission spectrum of the CDs aqueous solution under solar simulator irradiation is composed of the solar simulator output spectrum shaped by the absorption and emission of the CDs aqueous solution, confirming its ability to absorb and convert solar radiation. The photoluminescence characterization revealed a decrease in the emission intensity as the temperature was increased (Fig. 4a and S13† in the ESI) associated with the dynamic quenching due to the temperature enhanced population of non-radiative channels.^78,79^ The theoretical explanation behind this thermally activated non-radiative mechanism lies beyond the scope this work as the chemical/physical origin of the intrinsic emission in CDs remains an open question in the literature. Focusing on the best performing sample, a thermometric parameter^27–30^ was defined considering the total integrated area of the emission spectra (*S*_1_) and the average integrated intensity in the 480–490 nm spectral region (*S*_A_), similarly to what was previously reported for protein-based aqueous solutions:^16^4
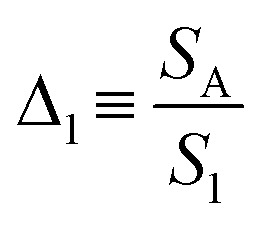


The ratiometric thermometric parameter is independent of sun irradiance fluctuations during a diurnal cycle and reveals a linear dependence on the temperature with a slope of (2.98 ± 0.04) × 10^−3^ °C^−1^ (Table 2 and Fig. 4c).

**Table 2:** Calibration curve slope (°C^−1^), thermal sensitivity (% °C^−1^) and temperature uncertainty (°C) of the *Δ*_1-2_ thermometric parameters

	Slope [°C^−1^]	*S*_r_ [% °C^−1^]	δ*T* [°C]
*Δ*_1_	(2.98 ± 0.04) × 10^−3^	0.33 ± 0.01	1
*Δ*_2_	(−8.60 ± 0.02) × 10^−2^	1.03 ± 0.03	0.4

The *S*_r_ value found was 0.33 ± 0.01% °C^−1^ (Table 2) which indicates the real possibility of having temperature monitoring.^29^ The δ*T* value found was 1 °C which indicates the possibility of accurately sensing temperature. We note that although *S*_r_ is commonly used as a figure of merit to compare different thermometers, it depends on the experimental conditions (*e.g.* emission spectra resolution) and on the sample characteristics, such as concentration and media.^28^ Nonetheless, we note that the values reported here are larger than that reported for an environmentally friendly nature-based thermometer based on enhanced green fluorescent protein (eGFP) (0.23% °C^−1^).^80^

As the photoluminescence spectra of the CDs aqueous solution are temperature-dependent (Fig. 4a), the optical and electrical performances of the LSC will also vary as a function of the temperature under solar simulator irradiation. This made us to analyse the electrical performance of the LSC-PV system as a function of the temperature. The generated electrical power (*P*_out_) decreases as the temperature is elevated (Fig. 4b and Table S3† in the ESI), allowing the definition of a new thermometric parameter:5
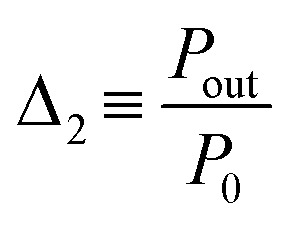
where *P*_0_ is the reference power value measured at room temperature (25 °C). The thermometric parameter follows a linear dependence with temperature (Fig. 4e), with a slope of (−8.60 ± 0.02) × 10^−2^ °C^−1^ (Table 2). The best *S*_r_ value was 1.03 ± 0.03% °C^−1^, with δ*T* of 0.4 °C, which guarantees the possibility of accurately sensing temperature (Table 2 and Fig. 4f). From the results presented above, it is worth noting that temperature affects two distinct parameters in the same device: the emission intensity and *P*_out_. We note that, according to the manufacturer, the thermal dependence of the power generated by bare PV cells is very low (<1%) in the considered temperature range.^81^ Since both established thermometric parameters *Δ*_1_ and *Δ*_2_ follow a linear dependence, they can be used for multiparametric thermal reading as they represent two independent pathways for temperature determination (multi-readout concept).^29^ This is a novel and very particular feature of the reported sensors, since this may impart an additional self-test functionality to the device.

## 4 Conclusions

In this work, luminescent solar concentrators (LSCs) with temperature sensing ability using highly efficient Rhodamine B-derived carbon dots (CDs) were reported. The CDs were dispersed in an aqueous medium and incorporated into organic–inorganic hybrid matrices, resulting in highly efficient materials (photoluminescent quantum yield values up to 82%) leading to optical conversion efficiency values up to 5.4%. The emission of these optically active materials showed temperature dependency, allowing the fabrication of solar optical temperature sensors, with thermometric parameters based on the emission and also on the electrical power generated by the LSC-PV system, with the maximum relative sensitivity of 1.0% °C^−1^. These results show that temperature plays a relevant role in the LSC performance, making it crucial to know the working temperature of the LSC during real use since it will impact the final performance of the device. Usually, results regarding LSC figures of merit under 1 sun illumination are reported as room temperature data, but typically, illumination could inherently increase the LSC temperature. Thus, the impact of temperature on the LSCs' overall performance during their experimental characterization cannot be neglected and should be included in standard protocols for LSCs characterization. In summary, this work is a starting point and validates the potential development of CD-based LSCs for future energy-efficient buildings and smart cities, emphasizing the need for procedure standardization to experimentally characterize LSC devices which cannot be underestimated by researchers working in this field.

## Author contributions

RASF and PSA conceived the idea of the experiment; experimental data were collected by SFHC and LMSD. The carbon dots production was developed and optimized by LF and characterized by TEM and XPS by RFPP and VZB. The sensor characterization was performed by PSA, SFHC and LMSD. RASF, PSA, SFHC and VZB co-wrote the paper with input from all authors. All authors have read and agreed to the published version of the manuscript.

## Conflicts of interest

There are no conflicts to declare.

## Supplementary Material

NA-005-D3NA00136A-s001
