# Supplementary material for: An autonomous power temperature sensor based on window-integrated transparent PV using sustainable luminescent carbon dots
Source: Nanoscale Adv. 2023 Apr 21;5(13):3428–38. doi: 10.1039/d3na00136a (PMC10295119; doi:10.1039/d3na00136a)
Supplement: NA-005-D3NA00136A-s001 [file NA-005-D3NA00136A-s001.pdf]

Electronic Supplementary Material (ESI) for Nanoscale Advances.
This journal is © The Royal Society of Chemistry 2023

Supporting Information

Autonomous power temperature sensor based on window-integrated transparent PV using sustainable luminescent carbon dots

Sandra F. H. Correia,*[a†] Lianshe Fu, [b†] Lília M. S. Dias,[b,c] Rui F.P. Pereira,[d] V. de Zea Bermudez,[e] Paulo S. André[c] and Rute A. S. Ferreira *[b]

[a]*Instituto de Telecomunicações and University of Aveiro, Campus Universitário de Santiago, 3810-193 Aveiro, Portugal.*

[b]*Department of Physics and CICECO - Aveiro Institute of Materials, University of Aveiro, 3810-193 Aveiro, Portugal.*

[c]*Department of Electrical and Computer Engineering and Instituto de Telecomunicações, Instituto Superior Técnico, University of Lisbon, 1049-001 Lisbon, Portugal.*

[d]*Chemistry Center and Chemistry Department, University of Minho, 4710-057 Braga, Portugal*

[e]*Chemistry Department and CQ-VR, University of Trás-os-Montes e Alto Douro, 5000-801 Vila Real, Portugal*

***Correspondence:** sandracorreia@ua.pt; rferreira@ua.pt

[†] These authors contributed equally to the work.

Contents:

S1. LSCs performance evaluation parameters.

Table S1. Details of the produced samples based on d-U(600) organic-inorganic hybrid doped with CDs and photoluminescence quantum yield (Φ) values measured under 510 nm excitation.

Table S2. Absolute emission quantum yield values (Φ) of RhB and CDs aqueous solution at distinct excitation wavelengths (λ_{exc}).

Table S3. Temperature-dependent output short-circuit current (I_{sc}), open-circuit voltage (V_{oc}) and power (P_{out}) of the PV cell coupled to the CDs aqueous solution LSC.

Fig. S1. (a) XRD patterns of NaCl, CDs and dU6/CDs-x, x=1, 2, 3, 4) and (b) FT-IR spectra of RhB, CDs and dU6/CDs-x (x = 1, 2, 3, 4).

Fig. S1. Room-temperature (a) excitation and (b) emission spectra of CDs aqueous solution.

Fig. S2. (a) Emission spectra of RhB aqueous solution with different concentrations, (b) the normalized emission spectra and (c) CIE 1931 chromaticity diagram.

Fig. S3. (a) Emission spectra of CDs aqueous solution with different concentrations, (b) the normalized emission spectra and (c) CIE 1931 chromaticity diagram.

Fig. S4. Room-temperature (a) excitation and (b) emission spectra of dU6/CDs-1.

Fig. S5. Room-temperature (a) excitation and (b) emission spectra of dU6/CDs-2.

Fig. S6. Room-temperature (a) excitation and (b) emission spectra of dU6/CDs-3.

Fig. S7. Room-temperature (a) excitation and (b) emission spectra of dU6/CDs-4.

Fig. S8. Reflectance curve of the reflective tape used on the LSCs.

Fig. S9. External quantum efficiency curve of the PV coupled to the LSC based on the CDs aqueous solution.

Fig. S10. (a) Absorbance and (b) transmittance spectra of the LSCs.

Fig. S11. (a) Spectra of the transmitted light of the LSCs under AM 1.5G solar simulator and (b) CIE 1931 color space diagram showing color coordinates of the LSCs.

Fig. S12. Temperature-dependent emission spectra of dU6/CDs-1 excited at (a) 330 nm, (b) 360 nm and (c) 470 nm.

Table S1. Details of the produced samples based on d-U(600) organic-inorganic hybrid doped with CDs and photoluminescence quantum yield (Φ) values measured under 510 nm excitation.

Sample	Amount of NaOH CDs solution (μL)	Φ
dU6/CDs-1	25	0.78±0.08
dU6/CDs-2	50	0.77±0.08
dU6/CDs-3	100	0.74±0.07
dU6/CDs-4	200	0.67±0.07

Table S2. Absolute photoluminescence quantum yield values (Φ) of RhB and CDs aqueous solution at distinct excitation wavelengths (λ_{exc}).

RhB concentration mg/L	λ_{exc} nm	Φ	CDs concentration mg/L	λ_{exc} nm	Φ (%)
0.48	553	30±3	0.43	488	82±8
4.79	534	23±2	4.25	496	81±8
47.9	400	12±1	42.5	446	57±6
479	445	2±1	425	524	27±3

Table S3. Temperature-dependent output short-circuits current (I_{sc}), open-circuit voltage (V_{oc}) and power (P_{out}) of the PV cell coupled to the CDs aqueous solution LSC.

T (°C)	I (×10^{-3} A)	V (V)	P_{out} (×10^{-4} W)
14.9	0.320	3.205	7.69
19.7	0.316	3.106	7.36
25.4	0.310	3.023	7.03
29.7	0.307	2.954	6.80
35.0	0.303	2.878	6.54
40.1	0.295	2.788	6.17
45.0	0.287	2.701	5.81

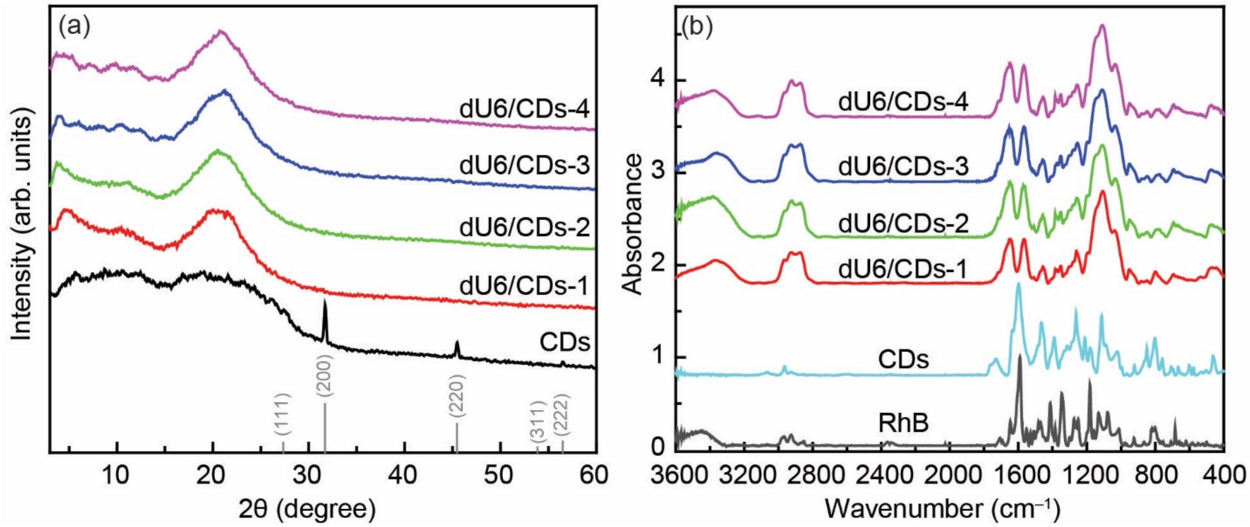

Fig. S1. (a) XRD patterns of NaCl, CDs and dU6/CDs-x, x=1, 2, 3, 4) and (b) FT-IR spectra of RhB, CDs and dU6/CDs-x (x = 1, 2, 3, 4).

For the XRD patterns of CDs (Fig. S1a), the broad peak centered at 21.6 (d = 0.44 nm), which is different from the crystalline structure of the pure RhB, is attributed to the (002) lattice spacing of graphite-like carbon-based materials.[1] The similar result to that of the literature indicates that the graphite-based carbon cores were formed. The sharp peaks located at 27.3, 31.7, 45.4, 53.9 and 56.5° are associated with NaCl, matching well with its PDF file (No. 05-0628), suggesting that some NaCl was encapsulated in the CDs solid when the basic CDs aqueous solution containing NaOH was neutralized by HCl. All the XRD patterns of dU6/CDs-x (x = 1, 2, 3 and 4) mainly show the bands related to the matrix d-U(600).

The FT-IR spectra of RhB, CDs, dU6/CDs-x (x = 1, 2, 3 and 4) are shown in Fig. S1b. The strong vibration bands at 1343 and 1467 cm^{-1} are characteristic of the stretching modes of the C–N– and $–CH_2–$ species of the RhB molecules, respectively.[2] The absorption bands that appeared at 1590 and 1467 cm^{-1} are assigned to the asymmetric and symmetric stretching vibrations of the COO− group.[3] The bands at 1343 and 1250 cm^{-1} are attributed to the vibrations from the aromatic skeletal C–C and C–O stretching. In addition, the band at 1710 cm^{-1} is assigned to C = N stretching vibration, while the shoulder at 1695 cm^{-1} is from the carbonyl group of RhB.[4] After the reaction, the intensities of these two bands decrease and a new band at 1730 cm^{-1} and a shoulder at 1763 cm^{-1} appear, indicating that carbonyl groups have changed during the thermal pyrolysis of RhB in NaOH aqueous solution. The vibration bands from CDs are not observed in the FT-IR spectra of dU6/CDs-x (x = 1, 2, 3 and 4) maybe due to the lower doping contents, for instance, around 0.1 wt% for dU6/CDs-4. To compare the photoluminescence properties between RhB and CDs aqueous solutions, the emission spectra of RhB and CDs with different concentrations are measured at room temperature, as shown in Fig. S2 and S3, respectively.

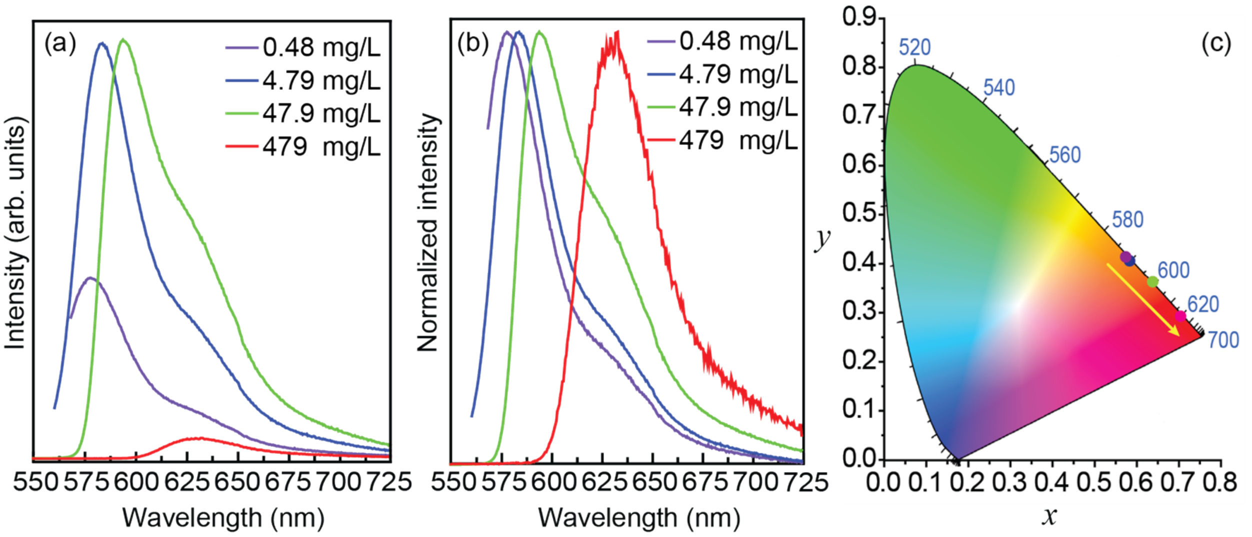

Fig. S2. (a) Emission spectra of RhB aqueous solution with different concentrations, (b) the normalized emission spectra and (c) CIE 1931 chromaticity diagram.

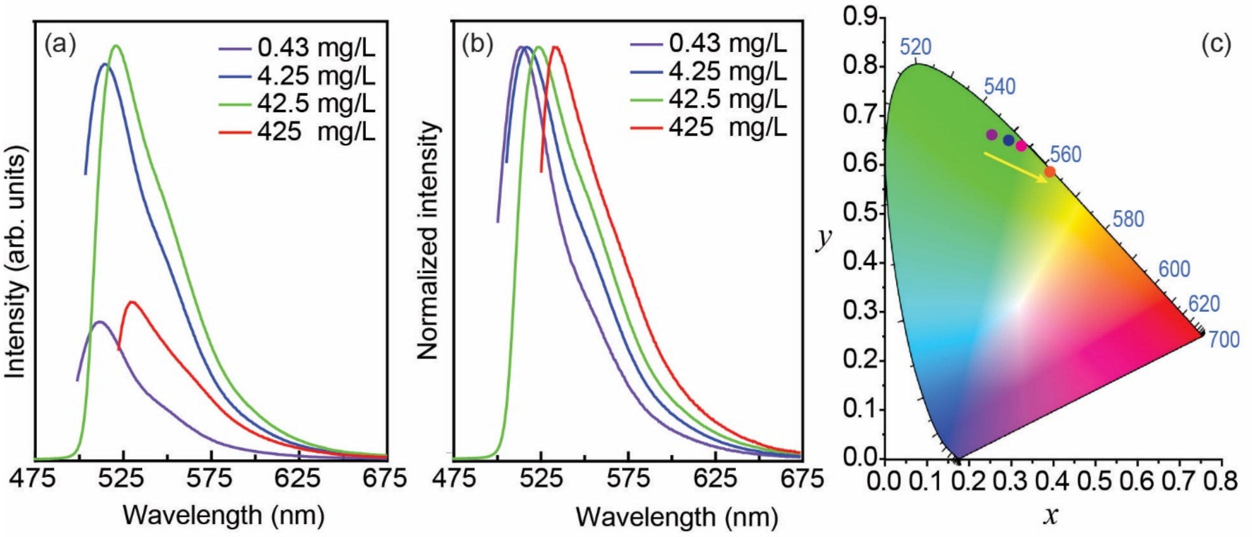

Fig. S3. (a) Emission spectra of CDs aqueous solution with different concentrations, (b) the normalized emission spectra and (c) CIE 1931 chromaticity diagram.

For RhB aqueous solution, the emission wavelength increases from around 580 to 630 nm with increasing of the concentration. The emission colors are mainly in the red region (Fig. S2c). The maximum Φ values under this study is 0.30±0.03 for 1×10^{-6} M RhB solution (Table S1). On the other hand, for CDs aqueous solution, the emission wavelength also increases with the increasing of the concentration, changing from around 515 to 535 nm, and the main emission color is in the green region (Fig. S3c). The Φ values can reach 0.82±0.08 for 0.43 mg/L CDs solution (Table S1).

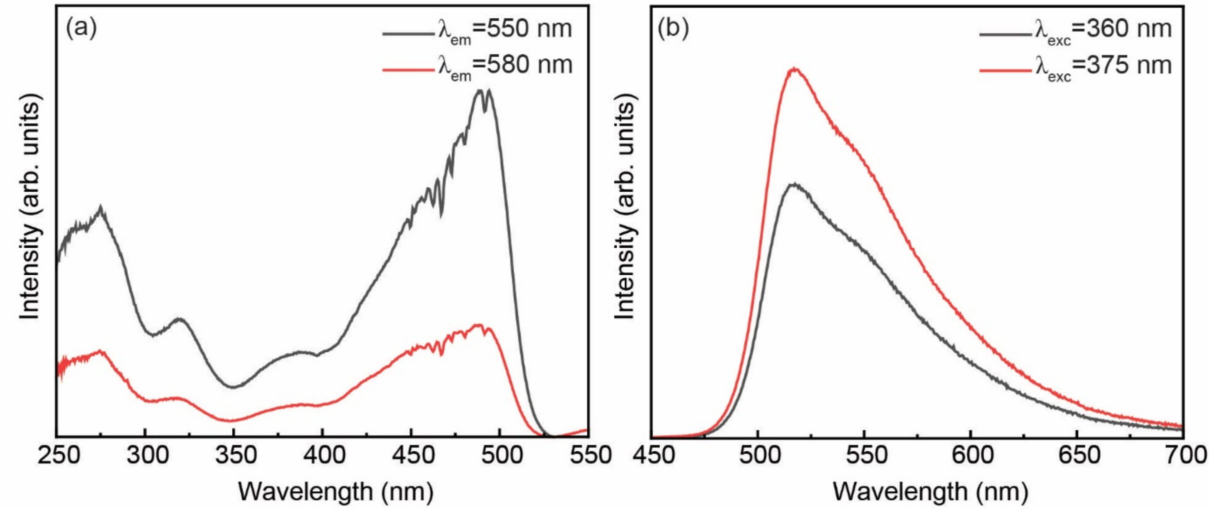

Fig. S4. Room-temperature (a) excitation and (b) emission spectra of CDs aqueous solution.

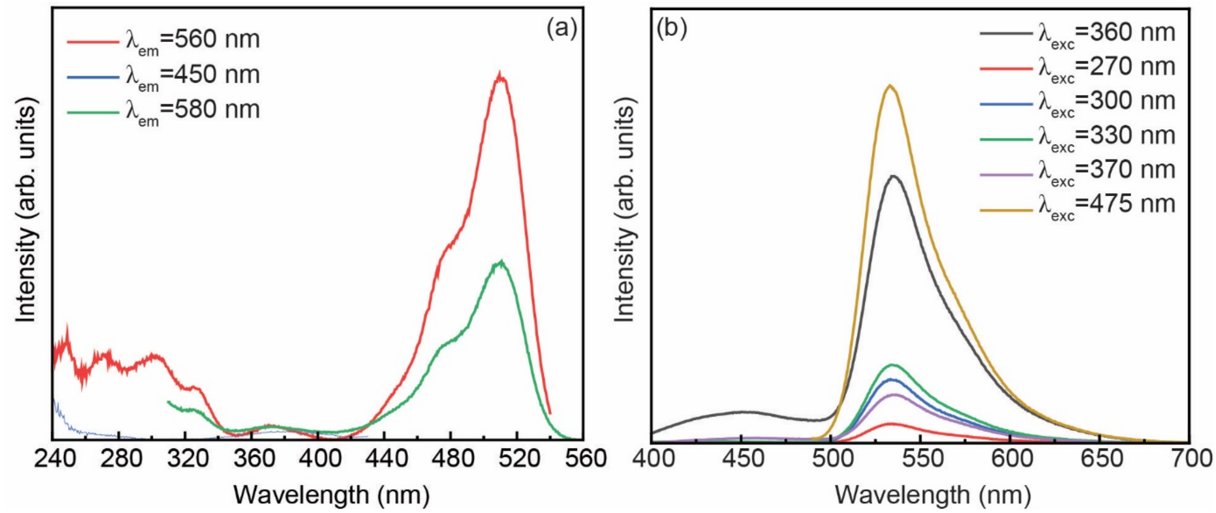

Fig. S5. Room-temperature (a) excitation and (b) emission spectra of dU6/CDs-1.

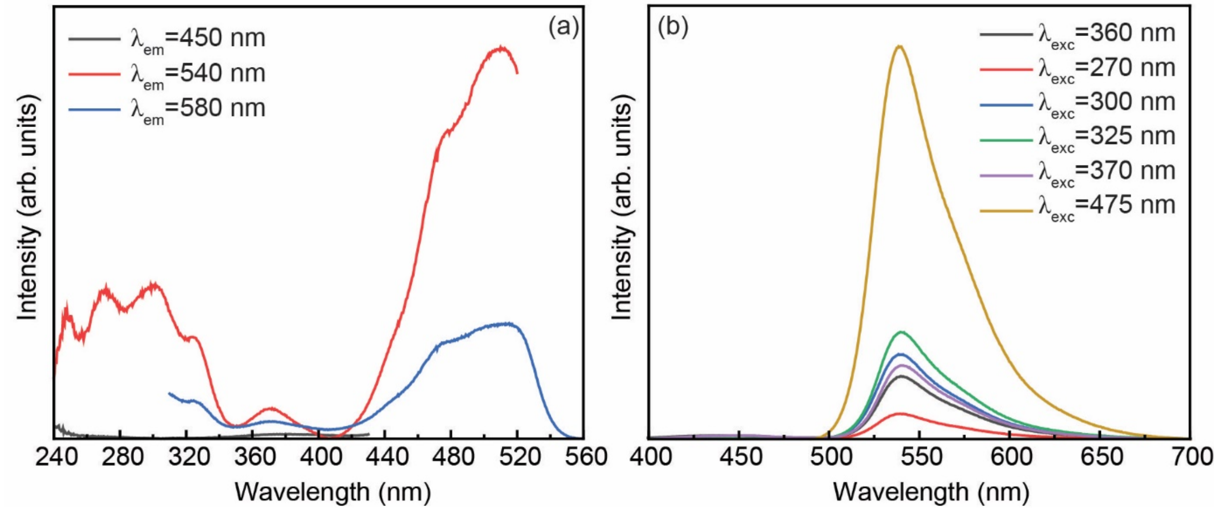

Fig. S6. Room-temperature (a) excitation and (b) emission spectra of dU6/CDs-2.

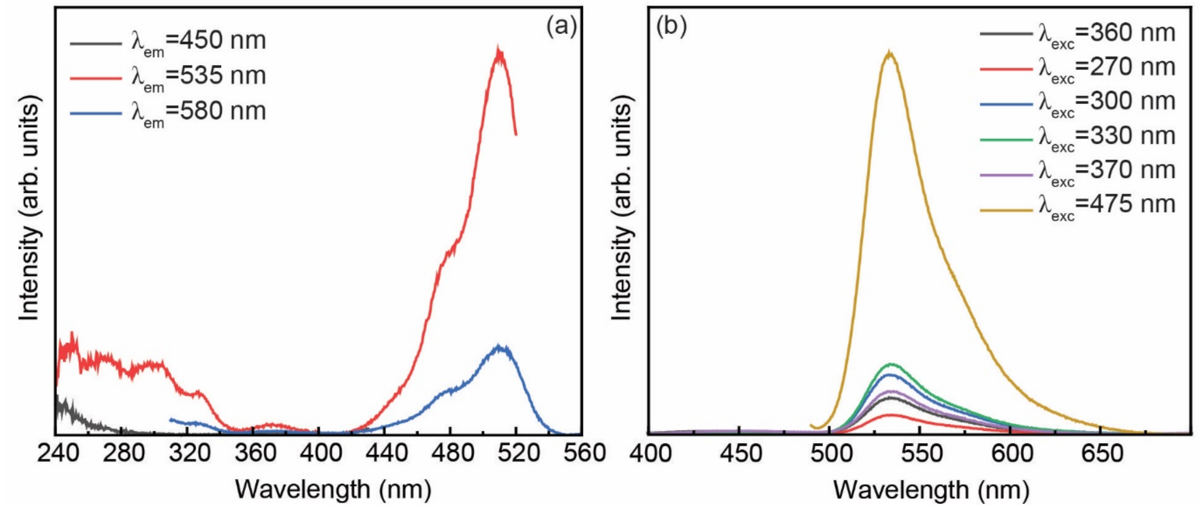

Fig. S7. Room-temperature (a) excitation and (b) emission spectra of dU6/CDs-3.

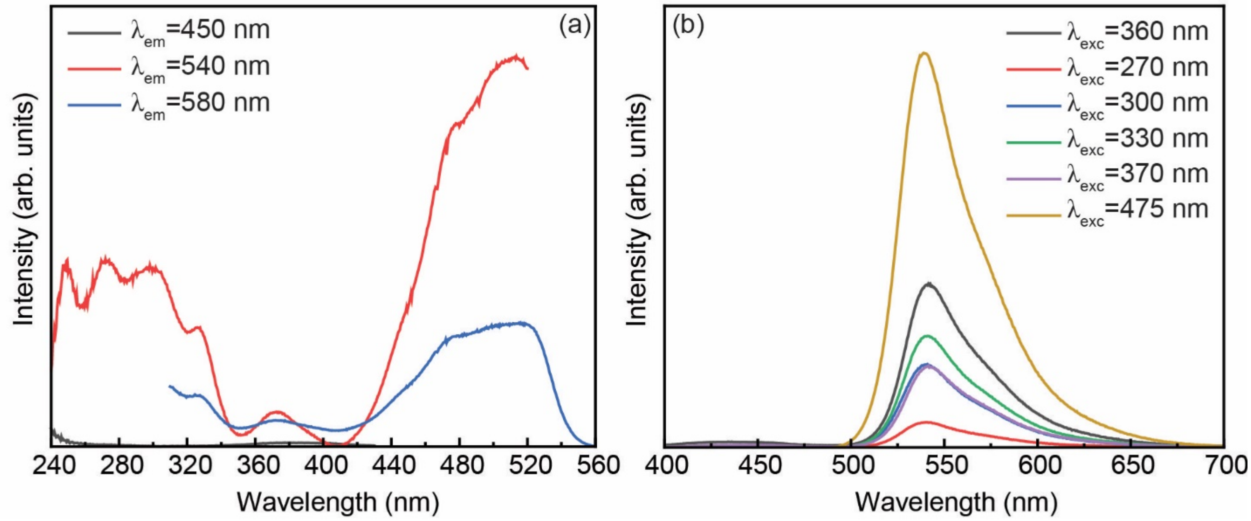

Fig. S8. Room-temperature (a) excitation and (b) emission spectra of dU6/CDs-4.

S1. LSCs performance evaluation parameters

The optical conversion efficiency (η_{opt}) was estimated by equation (S1):[5]

$$\eta_{opt} = \frac{P_{out}}{P_{in}} = \frac{I_{SC}^{L} V_{oc}^{L} A_e \eta_{solar}}{I_{SC} V_{oc} A_s \eta_{PV}} \quad \text{(S1)}$$

where I_{SC}^{L} and V_{oc}^{L} represent the short-circuit current and the open-circuit voltage when the PV device is coupled to the LSC, I_{sc} and V_{oc} are the corresponding values of the PV device exposed directly to solar radiation, A_s and A_e are the exposed and total edge areas respectively, η_{solar} is the efficiency of the PV device relative to the total solar spectrum and η_{PV} is the efficiency of the PV device at the LSC emission wavelengths. Three measurements were performed for each case, with a found relative error ($\eta_{opt}/\Delta\eta_{opt}$) below 10 %.

The experimental η_{opt} values were determined by illuminating the top surface of the LSCs (2.0×2.0 cm^2) with AM1.5G illumination from a solar simulator. The optical power at the LSC output was estimated using a commercial c-Si PV panel (KXOB22-01X8F, IXYS, composed of 8 cells) coupled to one edge of the LSC while the remaining edges were covered with reflective tape (Fig. S9). The PV cells have a total active area of 2.2x0.7 cm^2 closely matching that of the LSC edge (2.0×1.0 cm^2) to maximise light coupling. The I_{sc} and V_{oc} values were measured using a current source meter device (2400 SourceMeter SMU Instruments, Keithley). All measurements were performed under AM1.5G illumination (1000 $W \cdot m^{-2}$) using a 150 W xenon arc lamp, class A, solar simulator (Model 10500, Abet Technologies). The mismatch in the UV spectral region between the AM1.5G solar irradiance and that of the Xe lamp in the solar simulator was taken into consideration following a methodology reported in detail elsewhere.[6] Since the incident radiation from the solar simulator induces some heating of the LSCs, the external quantum efficiency (EQE) measurements were performed on the PV cell coupled to the LSCs with controlled temperature conditions and were calculated following equation (S2):

$$EQE(\lambda) = \frac{I_{SC} \cdot h \cdot c}{P_{in} \cdot e \cdot \lambda} \quad \text{(S2)}$$

where e is the charge of the electron, h is Planck's constant, c is the speed of light and λ is the wavelength. The solar simulator was coupled to a monochromator (Triax 180, Horiba Scientific). The I_{sc} and P_{in} values were measured using the above-mentioned current source meter and a c-Si calibrated photodiode (FDS1010, Thorlabs), respectively. Fig. 3b shows a correlation of the EQE curve of the PV cell coupled to the edge of the LSC based on RhB-CDs aqueous solution and its excitation spectrum, showing that both curves are correlated. This means that the photons arriving at the cell are generated by excitation and emission of the RhB-CDs optically active centers. The mesg surface plot resultsfrom the multiplication of both curves.

The power conversion efficiency (PCE) was calculated following equation (S3):

$$PCE = \frac{P_{out}^{el}}{P_{in}} = \frac{I_{SC}^{L} V_{oc}^{L} FF}{A_S \int_{\lambda_1}^{\lambda_2} I_{AM1.5G}(\lambda) d\lambda} \tag{S3}$$

where P_{out}^{el} and FF=0.75 are the PV device output electrical power and fill factor of the PV cell, respectively. The FF value was selected considering the available information by the manufacturer[7] and the same conservative value of FF was considered for all the calculated power values, even though it is reported that the fill factor increases slightly for low intensities.[8]

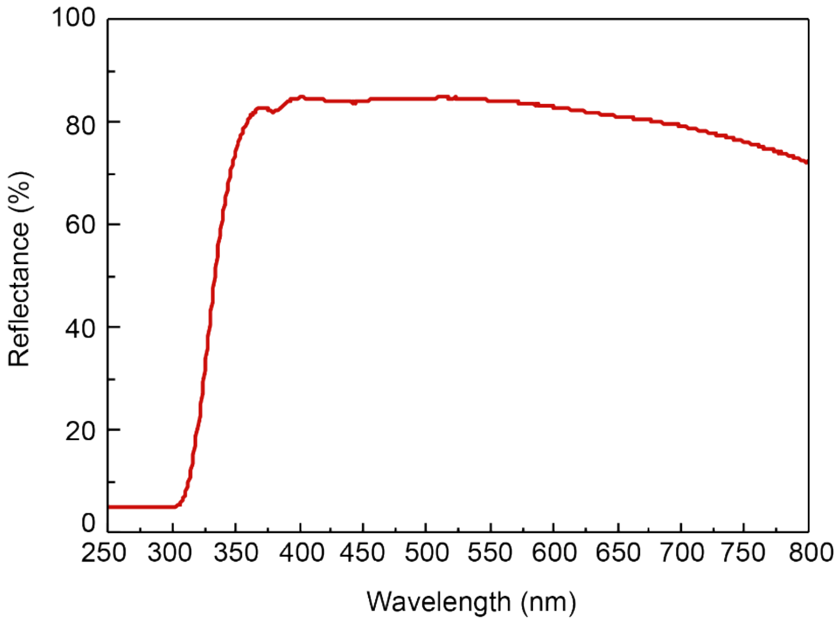

Fig. S9. Reflectance curve of the reflective tape used on the LSCs.

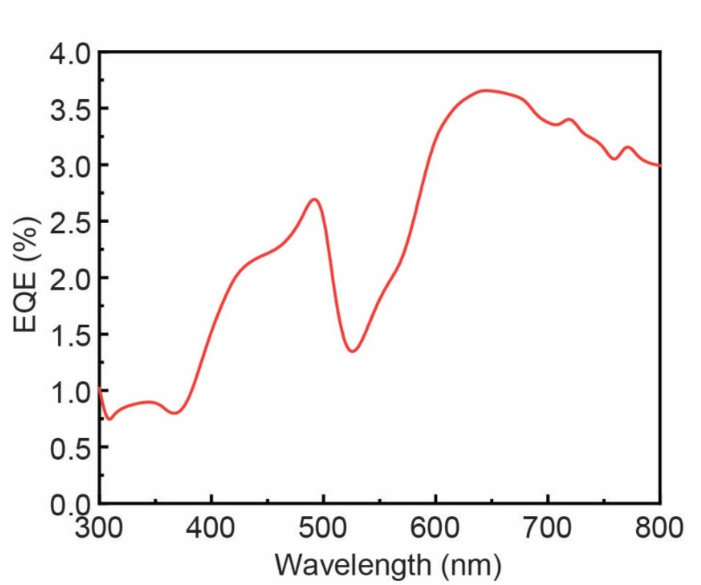

Fig. S10. External quantum efficiency curve of the PV coupled to the LSC based on the CDs aqueous solution.

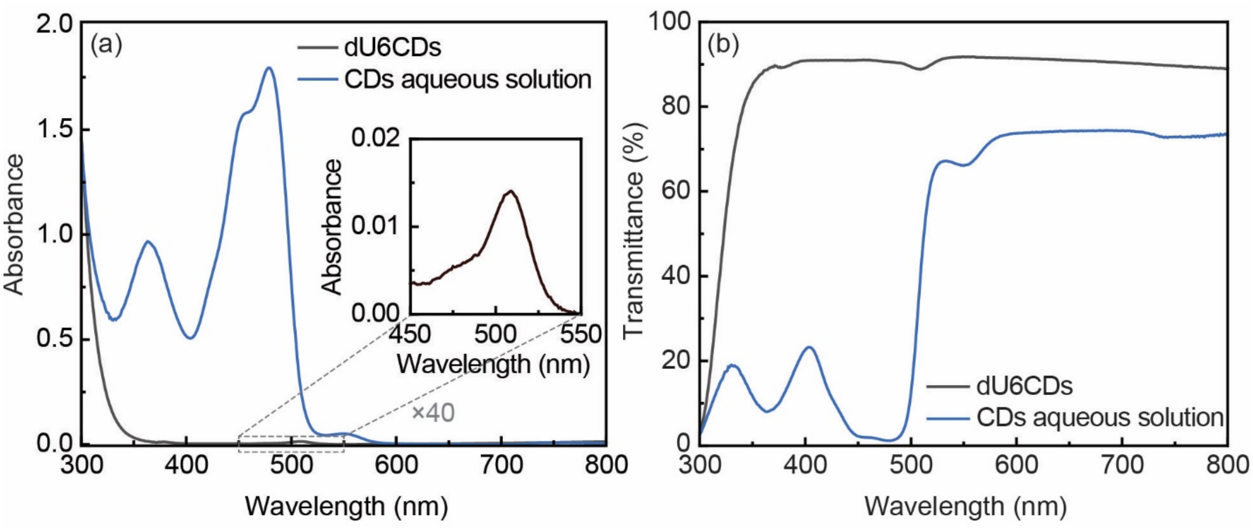

Fig. S11. (a) Absorbance and (b) transmittance spectra of the LSCs.

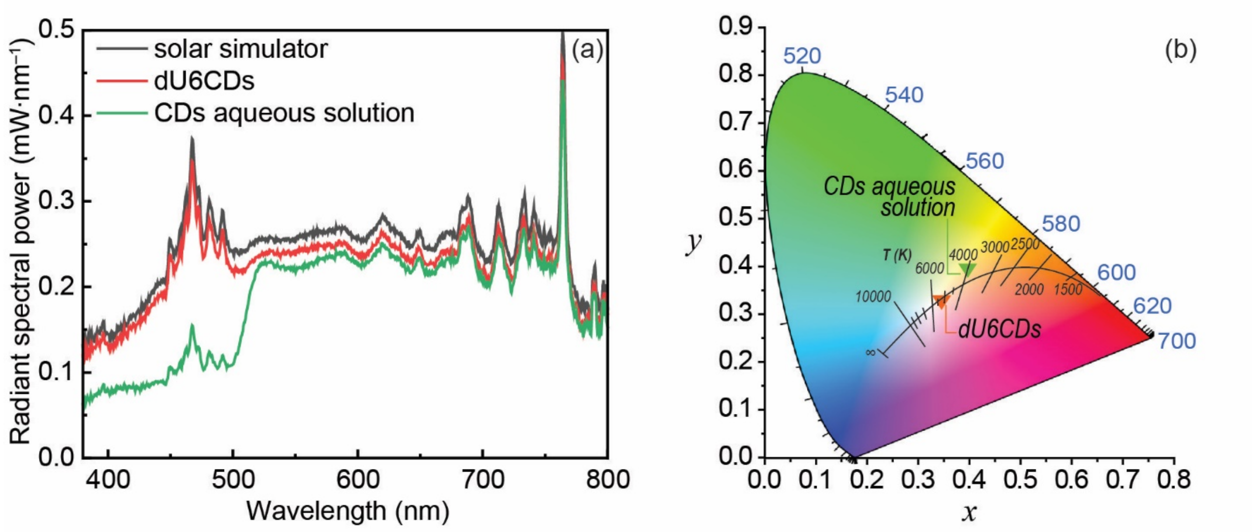

Fig. S12. (a) Spectra of the transmitted light of the LSCs under AM 1.5G solar simulator and (b) CIE 1931 color space diagram showing color coordinates of the LSCs.

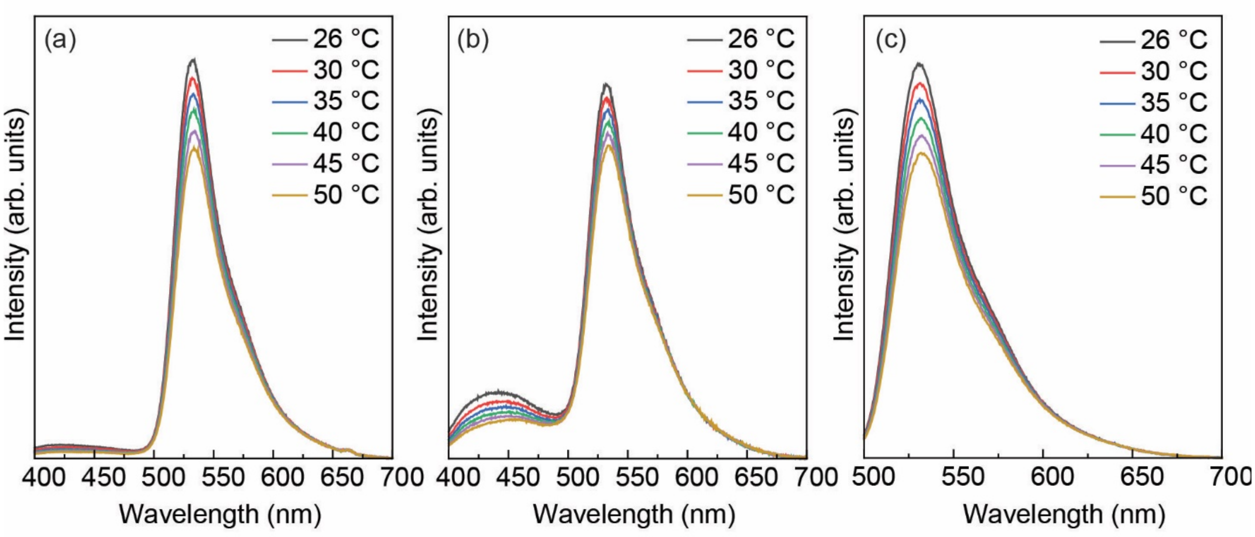

Fig. S13. Temperature-dependent emission spectra of dU6/CDs-1 excited at (a) 330 nm, (b) 360 nm and (c) 470 nm.

1. Z. G. Xu, X. B. Sun, P. P. Ma, Y. Q. Chen, W. Pan and J. P. Wang, *J. Mater. Chem. C*, 2020, **8**, 4557-4563.
2. A. Chouket, H. Elhouichet, R. Boukherroub and M. Oueslati, *Phys. Status Solidi A*, 2007, **204**, 1518-1522.
3. R. M. Dukali, I. M. Radovic, D. B. Stojanovic, D. M. Sevic, V. J. Radojevic, D. M. Jocic and R. R. Aleksic, *J. Serb. Chem. Soc.*, 2014, **79**, 867-880.
4. A. A. M. Farag and I. S. Yahia, *Opt. Commun.*, 2010, **283**, 4310-4317.
5. R. Reisfeld, D. Shamrakov and C. Jorgensen, *Sol. Energ. Mat. Sol. C*, 1994, **33**, 417-427.
6. S. F. H. Correia, P. P. Lima, P. S. Andre, R. A. S. Ferreira and L. D. Carlos, *Sol. Energ. Mat. Sol. C.*, 2015, **138**, 51-57.
7. IXOLAR™ High Efficiency SolarBIT IXYS KXOB22-01X8F, available at https://ixapps.ixys.com/DataSheet/KXOB22-01X8F_Nov16.pdf (accessed March 29th, 2023).
8. M. Chegaar, A. Hamzaoui, A. Namoda, P. Petit, M. Aillerie and A. Herguth, *Energy Proced.*, 2013, **36**, 722-729.
